# Editorial: Neutralizing Antibodies in the Prevention and Treatment of COVID-19

**DOI:** 10.3389/fimmu.2022.938069

**Published:** 2022-05-31

**Authors:** Raymund R. Razonable, Peter Chen

**Affiliations:** ^1^ Division of Public Health, Infectious Diseases and Occupational Medicine, Department of Medicine, Mayo Clinic, Rochester, MN, United States; ^2^ Department of Medicine, Women’s Guild Lung Institute, Cedars-Sinai Medical Center, Los Angeles, CA, United States

**Keywords:** SARS – CoV – 2, neutralizing antibodies, COVID-19 vaccines, COVID-19 Therapeutics, Convalescent plasma (CP) therapy

The COVID-19 therapeutic landscape rapidly evolved throughout the course of the SARS-CoV-2 pandemic. However, the ingenuity of our community quickly coalesced to identify novel ways to combat this deadly disease. One prime example is the infusion of anti-SARS-CoV-2 neutralizing antibodies as passive immunity to treat high-risk COVID-19 patients who are vulnerable to severe outcomes. Initially, convalescent plasma obtained from volunteers who have recovered from SARS-CoV-2 infections was used as a potential therapy for COVID-19 patients. Next, the rapid development of anti-SARS-CoV-2 monoclonal antibodies was added to our COVID-19 therapeutic armamentarium and was a stunning example of how contemporary technology can lead to discovery and validations of novel therapeutics. Neutralizing antibodies also play a key role in the immunity conferred by SARS-CoV-2 vaccines. Indeed, COVID-19 vaccination stimulates the production of high titers of neutralizing antibodies, which largely mediates the effect of infection prevention. Unfortunately, RNA viruses, such as SARS-CoV-2, are prone to mutations, and accordingly, SARS-CoV-2 variants have emerged that have developed resistance to neutralizing antibody therapies. As such, continued is needed to identify, develop and improve neutralizing antibodies in prevention and treatment of COVID-19. This Topic is a collection of pertinent studies that contribute to our understanding of the benefits of neutralizing antibody therapies in COVID-19 and develops tools for their improvement.

Convalescent plasma gained traction as a therapy for SARS-CoV-2 infections in the early days of the pandemic. The concept was that neutralizing antibodies in the plasma of individuals who have recovered from COVID-19 could be passively transferred to newly-infected individuals to reduce their viral load and alter the course of the infection towards clinical improvement and recovery. Initial retrospective cohort studies suggested benefit with the infusion of high-titer convalescent plasma particularly when given early in the hospital course. Subsequently, data from randomized controlled trials emerged as the pandemic progressed that muddled the support for its use as a therapy for those infected with SARS-CoV-2. Indeed, the meta-analysis by Jorda et al. finds that convalescent plasma had no benefit as a therapy in COVID-19. This conclusion was also reached by the NIH and IDSA (Infectious Disease Society of America) who do not recommend giving convalescent plasma to hospitalized COVID-19 patients. However, the US FDA currently continues to allow for the use of high titer convalescent plasma under emergency use authorization (EUA) for immunocompromised patients *early* in the course for COVID-19, even in the outpatient setting. Wirz et al. provides insight to the benefits of early use of convalescent plasma into patients infected with SARS-CoV-2; Only patients transfused before seroconversion, which *de facto* equates to early in the disease course, had demonstratable increase in plasma anti-SARS-CoV-2 antibody levels. Additionally, Yue et al. found the neutralizing ability of convalescent plasma is attenuated if collected prior to the emergence of variants of concern and is another consideration when choosing this treatment modality.

Monoclonal antibodies (mAb), another treatment from the passive transfer of neutralizing antibodies, were the next iteration of SARS-CoV-2 therapies that first obtained FDA EUA in November 2020. Lee et al. provide evidence for Regdanvimab as a mAb treatment to prevent progression to severe disease in high-risk patients. In the US, the FDA had granted emergency use authorizations for bamlanivimab, bamlanivimab-etesevimab, casirivimab-indevimab, sotrovimab, and bebtelovimab at various time points. However, their longevity has been short-lived because of the emergence of SARS-CoV-2 variants of concern (VOC) with mutations of the spike protein at the binding epitope can lead to resistance (Yue et al. and Tian et al.) Thus, the work by Shan et al. that identified a mAb that binds a conserved epitope in the receptor binding domain of the spike protein on the SARS-CoV-2 virus is pertinent due to the ability to potentially resist new VOCs that may emerge. Favorskaya et al. develop dimeric molecules that potently neutralize SARS-CoV-2 and decreases the chance of resistance to VOCs. Moreover, Mariotti et al. present a murine model to isolate and characterize mAbs that could help identify future mAbs for therapeutics and diagnostics. Additionally, Buratto et al. developed an *in silico* method to identify the binding affinity of the ACE2 receptor to the spike protein providing a platform to study SARS-CoV-2 mutations and help future development of neutralizing antibodies. A theoretical concern for mAbs was that treatment in SARS-CoV-2 infections would suppress the natural immunity from infection, but the findings of Zhang et al. dispelled this notion by showing sufficient maturation of anti-SARS-CoV-2 humoral immunity despite mAb treatment.

Finally, the development of vaccinations was another major breakthrough in our COVID-19 fight. Interestingly, Forgacs et al. show that the SARS-CoV-2 infection behaves as an antigenic boost that can augment the immunogenic response after COVID-19 vaccinations. The protective effect is largely mediated by the production of neutralizing antibodies, but several of the papers in this Topic discuss the need for standardized methods to evaluate efficacy that may not be fully represented by neutralizing antibody levels (Liu et al., Ravlić et al., Polvere et al.). The articles in this Topic highlights the collective efforts of our scientists around the world in developing antibody-based therapies for the prevention and treatment of COVID-19.

## Author Contributions

All authors listed have made a substantial, direct, and intellectual contribution to the work and approved it for publication.

## Funding

This work was supported by R01HL155759 (PC), R01HL137076 (PC), and an intramural grant at Mayo Clinic (RR).

## Conflict of Interest

Topic Editor RR received grants from Regeneron, Gilead, and Roche, for research purposes. Topic Editors PC received grants from Regeneron, Gilead, and Eli Lilly.

## Publisher’s Note

All claims expressed in this article are solely those of the authors and do not necessarily represent those of their affiliated organizations, or those of the publisher, the editors and the reviewers. Any product that may be evaluated in this article, or claim that may be made by its manufacturer, is not guaranteed or endorsed by the publisher.

